# The Acute Physiological and Perceptual Responses Between Bodyweight and Treadmill Running High-Intensity Interval Exercises

**DOI:** 10.3389/fphys.2022.824154

**Published:** 2022-03-10

**Authors:** Gabriella F. Bellissimo, Jeremy Ducharme, Zachary Mang, Desmond Millender, Jessica Smith, Matthew J. Stork, Johnathan P. Little, Michael R. Deyhle, Ann L. Gibson, Flavio de Castro Magalhaes, Fabiano Amorim

**Affiliations:** ^1^Health, Exercise and Sports Sciences, University of New Mexico, Albuquerque, NM, United States; ^2^School of Health and Exercise Sciences, University of British Columbia, Kelowna, BC, Canada; ^3^Department of Physical Education, Universidade Federal dos Vales do Jequitinhonha e Mucuri, Diamantina, Minas Gerais, Brazil

**Keywords:** high-intensity exercise, interval exercise, bodyweight exercise, calisthenics exercise, treadmill running exercise, acute exercise response

## Abstract

**Objective:**

The purpose of this study was to compare the acute physiological, perceptual, and enjoyment responses between bodyweight high-intensity interval exercise (BW-HIIE) and treadmill running high-intensity interval exercise HIIE (RUN-HIIE).

**Methods:**

Twelve adults [age: 29.5 ± 5.3 years; weight: 70.9 ± 15.0 kg; height: 167.9 ± 8.9 cm; peak oxygen consumption (VO_2_ peak): 48.7 ± 6.5 ml min^−1^·kg^−1^] performed both RUN-HIIE and BW-HIIE. RUN-HIIE consisted of two sets of 5, 60-s (s) run intervals at 100% of the speed achieved during VO_2_ peak testing followed by 60s of walking at 4.02 km/h. BW-HIIE consisted of two sets of 5, 60s ‘all-out’ effort calisthenic exercises followed by 60s of marching in place at 100 steps per minute. Oxygen consumption (VO_2_), blood lactate (B_lac_), heart rate (HR), and rating of perceived exertion (RPE) were measured during exercise. Physical activity enjoyment (PACES) was assessed post-exercise. Creatine Kinase (CK) was measured before exercise and 48-h post-exercise. Muscle soreness was assessed before exercise, post-exercise, and 48-h post-exercise.

**Results:**

Oxygen consumption relative to VO_2_ peak was higher (*p* < 0.001) during RUN-HIIE (88 ± 3%) compared to BW-HIIE (77 ± 4%). HR relative to HRpeak was higher (*p* = 0.002) for RUN-HIIE (93 ± 1%) compared to BW-HIIE (88 ± 2%). B_lac_ was higher (*p* < 0.001) after BW-HIIE (11.2 ± 3.2 mmol/l) compared to RUN-HIIE (6.9 ± 2.0 mmol/l). Average RPE achieved was higher (*p* = 0.003) during BW-HIIE (16 ± 2) than RUN-HIIE (14 ± 2). PACES was similar for RUN-HIIE and BW-HIIE (*p* > 0.05). No differences (*p* > 0.05) in CK were observed between RUN-HIIE and BW-HIIE.

**Conclusion:**

Our results indicate ‘all-out’ calisthenic exercise can elicit vigorous cardiorespiratory, B_lac_, and RPE responses. Implementing this style of exercise into training requires minimal space, no equipment, and may elicit cardiometabolic adaptations seen with traditional forms of high-intensity exercise.

## Introduction

Regular physical activity (PA) improves health, fitness, and quality of life, and reduces the risk of several chronic diseases and premature mortality (Booth et al., 2011). Major health organizations recommend achieving at least 150 min per week of moderate-intensity aerobic PA, or 75 min per week of vigorous-intensity aerobic PA, or any equivalent combination of the two, in addition to performing whole body muscle strengthening exercise at least twice per week (American College of Sports Medicine, 2018). Recent reports indicate only 23% of adults in the United States meet both the aerobic and muscle strengthening guidelines for PA (Blackwell and Clarke, 2018). The most common perceived barriers to PA include lack of time, access to exercise facilities, and the financial costs of exercise (Trost et al., 2002; Korkiakangas et al., 2009). Recent efforts to curb the spread of the Coronavirus Disease 2019 (COVID-19) (e.g., social distancing, remote work arrangements, and home isolation) have likely exacerbated barriers to PA and increased sedentary behaviors (Peçanha et al., 2020; Tison et al., 2020); this has shed light on the need for more studies assessing equipment-free and practical forms of exercise training.

High-intensity interval training (HIIT) has been shown to elicit similar cardiovascular and metabolic benefits as lower intensity, longer duration moderate-intensity continuous training (Burgomaster et al., 2008; Ryan et al., 2020); therefore, HIIT is often regarded as a time-efficient way to exercise. However, traditional HIIT exercises may require specialized equipment (i.e., treadmill and cycle ergometer) and/or facilities (i.e., gyms and parks). Bodyweight (BW) training exercises (e.g., squats, lunges, and burpees) do not require specialized equipment and can be performed using minimal space. Recently, Archila et al. (2021) reported that 11 min of BW interval exercises performed at an ‘all-out’ effort enhanced peak oxygen uptake (VO_2_peak) in healthy physically inactive adults after six weeks of training (Archila et al., 2021). In addition, Schaun et al. (2018) demonstrated that sixteen weeks of ‘all-out’ BW exercise-induced similar increases in VO_2_peak as HIIT performed on a treadmill (Schaun et al., 2018). Therefore, ‘all-out’ BW exercise may be regarded as a practical and effective form of HIIT for improving cardiorespiratory fitness.

High-intensity interval exercise (HIIE) is traditionally characterized by brief periods of vigorous exercise performed at “near maximal” efforts and intensities which elicit ≥85% of maximum heart rate (%HRmax) and/or ≥ 80% of maximum oxygen consumption (%VO_2_max), separated by periods of active or passive recovery (Helgerud et al., 2007; MacInnis and Gibala, 2017). According to the American College of Sports Medicine (ACSM), vigorous aerobic exercise can also be classified as 64–90% VO_2_max, 77–95%HRmax, and a rating of perceived exertion of somewhat hard to very hard (RPE 14–17; Garber et al., 2011; American College of Sports Medicine, 2018). Less data exist regarding the cardiorespiratory and perceptual characteristics associated with calisthenic intervals performed at an ‘all-out’ effort; however, previous studies suggest this style of exercise can be considered a form of vigorous aerobic exercise. For example, Gist et al. (2014) compared Wingate based cycling to ‘all-out’ repeated burpees and found the cardiorespiratory strain relative to peak values were similar between the protocols (Wingate: ~80.4% VO_2_peak and ~ 86.8% HRpeak versus burpees: ~77.6%VO_2_peak and ~ 84.6% HRpeak; Gist et al., 2014). Further, both protocols elicited significant increases in blood lactate (B_lac_; Wingate: ~9.1–12.8 mmol/l versus burpees: ~3.7–8.3 mmol/l) and were ranked as “hard” to “very hard” on the RPE scale (Gist et al., 2014). Riegler et al. (2017) compared 30 s (s) of cycle ergometry intervals performed at 70% of peak power output to a popular smartphone protocol comprised of calisthenics performed for as many repetitions as possible in 30s (i.e., jumping jacks, wall sit, push-ups, abdominal crunches, step-ups onto chair, squats, triceps dips on chair, planks, high knees, lunges, push-up with rotation, and side planks; Riegler et al., 2017). This mix of calisthenic exercises elicited ~90%HRmax, ~81%VO_2_max and B_lac_ levels >5 mmol/l (Riegler et al., 2017). The pioneering studies mentioned above (Gist et al., 2014; Riegler et al., 2017) suggest calisthenics performed at an ‘all-out’ effort may elicit slightly lower cardiorespiratory strain than traditional forms of HIIE; however, they can be classified as vigorous exercise based on the aforementioned ACSM classifications. Further, both Gist et al. (2014) and Riegler et al. (2017) report B_lac_ levels >4 mmol/l which is traditionally associated with the onset of blood lactate accumulation (Heck et al., 1985) and levels observed in studies assessing the acute responses of traditional forms of HIIE (Wood et al., 2016; Olney et al., 2018).

The greater eccentric components involved with ‘all-out’ bodyweight high-intensity exercise (BW-HIIE) may result in more contraction-induced muscle damage compared to traditional forms of HIIE such as cycle ergometry or treadmill running (Lieber and Fridén, 1999). Exercise-induced muscle damage may include sarcolemmal disruption and consequently the appearance of sarcoplasmic proteins [i.e., creatine kinase (CK)] in the blood and may also be associated with delayed-onset muscle soreness (Clarkson and Hubal, 2002). This unpleasant sensation resulting from BW-HIIE may discourage individuals who are unaccustomed to exercise from continuing an exercise regimen. In contrast, BW-HIIE may be an appealing alternative to the monotony of traditional HIIE due to the greater variety of exercises and movements. Moreover, BW-HIIE may be superior to traditional forms of HIIE due to its potential effectiveness at improving both cardiorespiratory fitness and muscular endurance (McRae et al., 2012). To understand these possible positive and negative effects of BW-HIIE, it is important to compare the acute cardiorespiratory, metabolic, and perceptual responses to traditional weight bearing forms of HIIE, such as treadmill running.

The purpose of the present study was to compare the acute physiological (VO_2_, HR, B_lac_, and blood CK concentration) and perceptual responses (RPE, muscle soreness, and enjoyment) between ‘all-out’ BW-HIIE to constant load treadmill running HIIE (RUN-HIIE). To our knowledge, no studies have compared predominantly lower body, plyometric-based calisthenic exercises (i.e., high knees, squat jumps, scissor jacks, jumping lunges, and modified burpees) to a traditional RUN-HIIE protocol. This combination of calisthenic exercises may elicit more comparable metabolic and cardiorespiratory responses to RUN-HIIE because both protocols combine high-intensity movements with weight bearing load. Further, limited evidence exists on the skeletal muscular strain responses of BW-HIIE (i.e., CK and muscle soreness). Understanding such responses will facilitate a better characterization of practical, cost-free forms of HIIE. In this study, we used a HIIE protocol (10 × 60s high-intensity intervals separated by 60s of active recovery), which has been previously shown to be efficacious and practical in healthy (Little et al., 2010) and clinical populations (Francois and Little, 2015). We hypothesized the plyometric exercises used in the BW-HIIE protocol would elicit lower cardiorespiratory responses (VO_2_ and HR) than RUN-HIIE but greater RPE, B_lac_, CK, and muscle soreness. We also predicted BW-HIIE would be considered more enjoyable than RUN-HIIE given our sample of cross-trained individuals.

## Materials and Methods

### Participants

Fourteen physically active adults were recruited from the local community; however, two withdrew participation due to injuries unrelated to the study. The total sample size was determined by *a priori* analyses using G^*^Power software (version 3.1.9.2) based on the following parameters: a statistical test of two dependent mean samples, an alpha level of 0.05, a power (1-beta) equal to 0.95, and an effect size of 1.055. The main variable of interest (VO_2_) was used to guide the power analysis based on the VO_2_ responses between self-propelled interval running versus bodyweight circuit intervals in adults who regularly exercised reported in a study by Schleppenback et al. (2017). All participants were considered physically active by meeting both aerobic (≥150 min of moderate-intensity or 75 min of vigorous-intensity/week) and strength training (≥2 days/week) recommendations (American College of Sports Medicine, 2018). All participants reported being accustomed to calisthenics, plyometrics, and high-intensity exercise through their training regimens (i.e., CrossFit, recreational sport, and circuit/resistance training). Participants were deemed healthy based on responses to a customized health history questionnaire. All participants gave written informed consent prior to participating. This study was approved by the local university institution review board.

### Baseline Testing

Participants’ height (cm) was measured using a stadiometer (Holtain Limited, Crymych, Dyfed, Great Britain) and weight (kg) was recorded using a digital weight scale (MedWeight MS-3900, Itin Scale Company, Brooklyn, NY, United States). Body composition was assessed using 3-site skinfold measurements and equations for men (chest, abdomen, and thigh; Jackson and Pollock, 1978) and women (triceps, suprailiac, and thigh; Jackson et al., 1980). The resulting body density was converted to body fat percent (%BF) using the Siri (1956) equation (Siri, 1956). All anthropometric data are shown in Table 1.

**Table 1:** Anthropometric and physiological data for female (*n* = 6) and male (*n* = 6) participants.

	Female (*n* = 6)	Male (*n* = 6)	All (*N* = 12)
Age (years)	30.5 ± 6.7	28.5 ± 3.7	29.5 ± 5.3
Height (cm)	161.8 ± 5.7	173.9 ± 7.5	167.9 ± 8.9
Weight (kg)	60.5 ± 5.1	81.3 ± 14.5	70.9 ± 15.0
BF (%)	20.1 ± 3.4	10.0 ± 3.8	15.0 ± 6.3
VO_2_peak (mL· min-^1^·kg-^1^)	43.2 ± 3.6	54.3 ± 3.4	48.7 ± 6.7

*N* = 12. cm, centimeters; kg, kilograms; BF (%), body fat percentage; VO_2_peak, peak oxygen consumption; and ml, milliliters, min, minute. Data are displayed as means ± standard deviation.

A VO_2_peak test was performed on a motorized treadmill (C966i, Precor Inc., Woodinville, WA, United States) while wearing a HR monitor around the chest (Garmin HRM, Olathe, KS, United States). Participants wore a nose clip while expired gases were collected using a two-way valve and mouthpiece (Hans Rudolph Inc. Kansas City, MO). Breath-by-breath sampling of expired gases was measured continuously by a metabolic cart (Parvo Medics True One 2,400, Sandy, UT, United States). The same metabolic cart and procedures were used for both RUN-HIIE and BW-HIIE trials. Before each test, the metabolic cart was calibrated according to manufacturer guidelines. Participants performed a 5 min warm-up while being instructed to find a pace they could maintain for 30+ minutes. This pace was selected as the initial starting pace for the test. All VO_2_peak tests were performed at a constant 3.0% grade while speed was increased by 0.8 km/h every minute until participants reached volitional fatigue. All VO_2_peak tests were completed within 8–12 min (Yoon et al., 2007) and defined by meeting a minimum of two of the following criteria: respiratory exchange ratio (RER) ≥ 1.1, maximal heart rate within 10 beats of calculated age-predicted HRmax; 208—(0.7 × age; Tanaka et al., 2001) or RPE ≥ 17 (Astorino et al., 2000; Robergs et al., 2010), without investigation of plateau. Metabolic data were smoothed using an 11-breath rolling average and the highest data point was recorded as VO_2_peak (ml·min^−1^ kg ^−1^; Robergs et al., 2010). Maximal velocity (Vmax) for the RUN-HIIE trial was determined by using the final speed achieved during the VO_2_peak test. If participants did not last at least 40 s in the final stage, the speed from the previous stage was recorded as Vmax. Five of the twelve participants were prescribed the previous speed achieved during VO_2_peak testing as their Vmax. VO_2_peak results are shown in Table 1. After baseline testing, participants were familiarized with the BW-HIIE protocol and were provided a document with images and instructions describing each movement.

### Study Design

**Figure 1:**
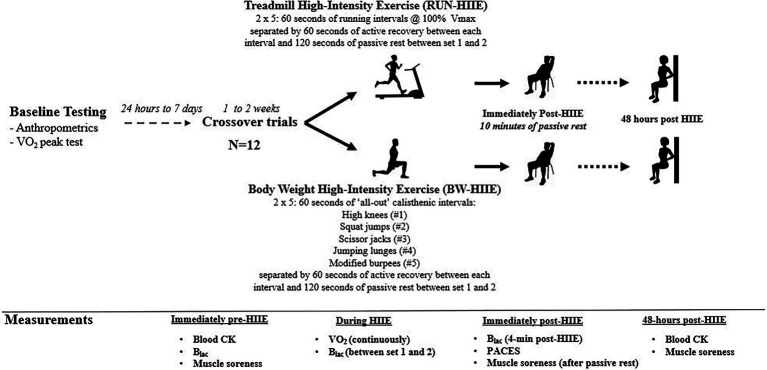
Summary of study design. RUN-HIIE, treadmill high-intensity exercise; BW-HIIE, body weight high-intensity exercise; VO_2_peak, maximal oxygen consumption; V_max_, maximal running velocity achieved during VO_2_peak test; CK, creatine kinase; B_lac_, blood lactate; VO_2_, oxygen consumption; and PACES = physical activity enjoyment scale.

The current study utilized a randomized counterbalanced crossover design where participants performed two HIIE trials (RUN-HIIE and BW-HIIE) separated by ≥7 days. The two HIIE trials were performed on different days but at a similar time of the day (within 1 to 2 h difference). Participants were asked to avoid performing vigorous exercise ≥24 h, refrain from caffeine consumption ≥4 h, and arrive fasted for ≥1 h before each trial. Each HIIE trial lasted 22 min (with the exclusion of warm-up). Both RUN-HIIE and BW-HIIE protocols consisted of two sets of five 60s intervals separated by 60s of active recovery and 120 s of passive recovery between the first and second sets. Measurements obtained for each HIIE trial included as: blood samples for CK, VO_2_, HR, B_lac_, RPE, physical activity enjoyment scale (PACES), and perceived muscle soreness. The study design and measurement time points are summarized in Figure 1.

### Run-HIIE Protocol

Before starting RUN-HIIE, participants performed a self-paced, 5 min warm-up of treadmill running. For the RUN-HIIE protocol, participants ran for 60s at 100% of the speed achieved during VO_2_ peak testing (V_max_), followed by 60s of walking at 4.02 km/h at a constant grade of 3% on a motorized treadmill (C966i, Precor Inc., Woodinville, WA, United States). Participants were provided the option of reducing the speed during RUN-HIIE if they felt unable to complete the 60s interval at V_max_; however, all participants were able to complete the intervals at their prescribed speed for both sets of RUN-HIIE. Participants were provided verbal encouragement throughout each RUN-HIIE bout.

### BW-HIIE Protocol

Before starting BW-HIIE, participants performed a self-paced, 5 min warm-up of calisthenic exercises led by a member of the research team (i.e., jogging in place, heel to glute kicks, high knees, ski jumps, and dynamic stretches). For the BW-HIIE protocol, participants performed five calisthenic exercises for as many repetitions as possible in 60s including: (1) high knees, (2) squat jumps, (3) scissor jacks, (4) jumping lunges, and (5) modified burpees (no push-up), followed by 60s of marching in place to a metronome cadence of 100 steps per minute. The BW-HIIE exercises and execution sequence were selected to alternate between aerobic type exercises (i.e., high knees, scissor jacks, and burpees) and muscle strengthening type exercises (i.e., squat jumps and jumping lunges). Participants were instructed to resort to modified versions (elimination of plyometric components) if they felt unable to complete the 60s. All participants were successful in completing the 60s of BW-HIIE; however, each participant resorted to the modified versions for squat jumps and jumping lunges to complete the 60s. No modifications were needed for the other three calisthenic exercises (high knees, scissor jacks, and modified burpees). Repetitions achieved for each exercise were recorded using a four-digit hand tally counter. For exercises with alternating movements (i.e., high knees, scissor jacks, and jumping lunges), repetitions were recorded for each leg separately. Squat jump repetitions were recorded as a break of ≥90 degrees at the hip for the decent position and a full hip extension for the accent position. Modified burpee repetitions were counted after a full completion of a squat into a plank position with straight arms, feet back to the squat position, and extension of the knees/hips while jumping with arms extended over the head. A member of the research team was responsible for holding the tubing connected to the metabolic cart while participants performed the BW-HIIE. Participants reported that the tubing did not interfere with their performance. Participants were provided verbal encouragement throughout each BW-HIIE bout.

### Blood Analyses, Data Processing, and Calculations

#### B_lac_ and CK

Blood samples (approximately 10 ml) were collected immediately before the warm-up and 48 h after each HIIE trial. All samples were collected in K2 EDTA tubes (Fisher Scientific, Carlsbad, CA) and centrifuged at 22^o^ C for 15 min at 3,950 *g* (Allegra X-14R Centrifuge, Beckman Coulter, Brea, CA). Plasma was separated into Eppendorf vials, immediately frozen, and stored at −80°C. When all blood samples had been collected and processed, the samples were sent to a commercial laboratory (QuestDirect^™^, Albuquerque, NM, United States) and analyzed for CK. Blood lactate (B_lac_) was obtained in duplicates from the earlobe using a handheld lactate meter (Lactate Plus, NOVA, Biomedical, MA); averages were recorded for all time points (i.e., rest, immediately after the first 5 intervals (post-set 1) and 4 min after the last 5 intervals (post-set 2)).

#### Oxygen Consumption and Heart Rate

Indirect calorimetry was used to measure metabolic gases continuously throughout each of the HIIE trails. All VO_2_ data from the HIIE trials were smoothed using the same method used for VO_2_peak testing (i.e., 11-breath rolling average). The highest value of relative VO_2_ (ml·min^−1^·kg^−1^) was defined as participants’ VO_2_peak for RUN-HIIE and BW-HIIE. Average VO_2_ for RUN-HIIE and BW-HIIE were expressed as %VO_2_peak. Heart rate (HR) was measured (Polar HR 600, Polar Electro Inc., Lake Success, NY, United States) and recorded immediately after each high-intensity interval for each of the HIIE trials. Heart rate relative to peak HR (%HRpeak) and average HR achieved during each high-intensity interval was calculated.

#### RPE, Perceived Muscle Soreness, and PACES

Rating of perceived exertion (RPE) was recorded immediately after each high-intensity interval for each of the HIIE protocols. The average and peak RPE for the high-intensity intervals were calculated for each HIIE protocol. Participants rated their leg muscle soreness using a 100 mm visual analog scale (0 = no soreness and 100 = the worst possible soreness) while performing a wall sit with their knees flexed 90 degrees (Fortes et al., 2013), before exercise, post-exercise (immediately after 10 min of passive rest), and 48 h after each of the HIIE trials. Average muscle soreness was determined by measuring the distance between what was marked by the participant and 0 mm mark for each of the HIIE protocols. Exercise enjoyment was determined by the PACES survey (Kendzierski and DeCarlo, 1991) and was administered during the 10 min of post-HIIE passive rest. Negatively worded PACES items were reverse-scored and scores for all 18 items were summed to calculate a total score (out of 126). The internal consistency for PACES was acceptable at each administration (Chronbach’s α ≥ 0.932).

### Statistical Analyses

All data are expressed as mean ± SD. Data were checked for normality and homogeneity of variance with the Shapiro–Wilk and Levene tests, respectively. The alpha level was set at a value of p of ≤0.05 for indication of statistical significance for all analyses. Two-tailed paired sample *t*-tests were used to identify statistical differences in measurements of RPE and PACES between conditions, and number of repetitions achieved (i.e., set 1 versus set 2) for BW-HIIE. Two-factor (condition × time) repeated measures ANOVA was used to examine the effects type of HIIE and time on %VO_2_peak achieved, %HRpeak, average HR, B_lac_, perceived muscle soreness, and CK concentration. Bonferroni’s correction factor was employed to test multiple comparisons. Partial eta squared ( $\eta_{p}^{2}$ ) was calculated to determine the magnitude of main and interaction effects and was categorized as small (0.01), medium (0.06), and large (0.14), respectively (Cohen, 1992; Lakens, 2013). Hedges’s *g* (*g*) was calculated for between and within group pairwise comparisons and defined as trivial (< 0.2), small (≥ 0.2 and ≤ 0.49), moderate (≥ 0.5 and ≤ 0.79), and large (≥ 0.8), respectively (Cohen, 1992; Lakens, 2013). All data were analyzed using STATISTICA (STATISTICA Software version 10 Tulsa, OK), except for *a priori* power analyses which was performed using G^*^Power 3 software (G^*^Power, version 3.1.9.2).

## Results

Table 2 depicts the number of repetitions performed during the BW-HIIE. Besides squat jumps, for which participants, on average, completed fewer repetitions during set 2 compared to set 1, *p* = 0.022, no significant differences (*p* ≥ 0.05) were observed in the number of repetitions achieved. Average V_max_ for set 1 and 2 of RUN-HIIE was 14.8 ± 1.8 km/h.

**Table 2:** Repetitions performed during BW-HIIE (*N* = 12).

Exercise	Set 1	Set 2	Value of *p*	*g*
High knees	185 ± 16	183 ± 22	0.770	0.100
Squat jumps	50 ± 6	47 ± 8	0.022^*^	0.410
Scissor Jacks	154 ± 13	153 ± 10	0.481	0.083
Jumping Lunges	55 ± 10	51 ± 11	0.527	0.367
Modified Burpees	24 ± 3	22 ± 4	0.076	0.546

*g* = Hedges’s *g*.

* *p* < 0.05 significantly different between sets.

### %VO_2_peak

**Figure 2:**
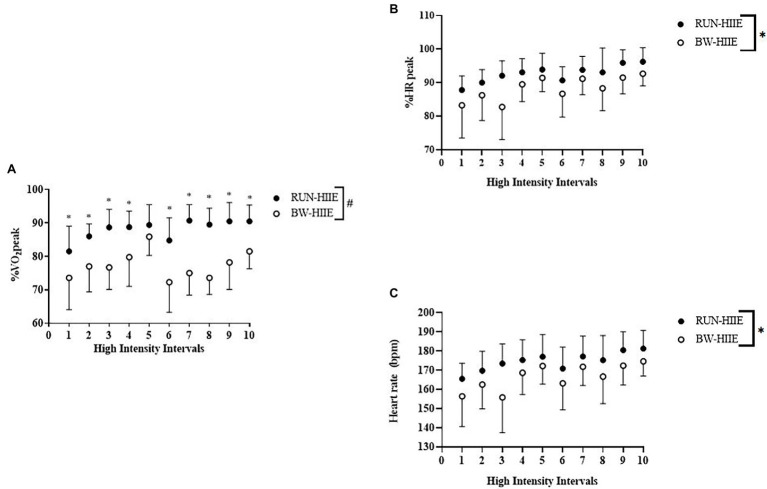
**(A)** High-intensity intervals 1–10 on %VO_2_peak for BW-HIIE and RUN-HIIE. **(B)** High-intensity intervals 1–10 on %HRpeak for BW-HIIE and RUN-HIIE. **(C)** High-intensity intervals 1–10 on average heart rate during RUN-HIIE and BW-HIIE. BW-HIIE, high-intensity interval exercise with bodyweight, RUN-HIIE, high-intensity interval exercise with treadmill, ^#^*p* < 0.05 RUN-HIIE was significantly higher than BW-HIIE, and ^*^p < 0.05 RUN-HIIE was significantly higher during interval than BW-HIIE. Data are expressed as mean ± SD.

Figure 2A displays the effect of high-intensity intervals 1–10 on average %VO_2_peak for BW-HIIE and RUN-HIIE. A significant interaction effect was observed on %VO_2_peak for type of HIIE exercise and sequence of exercise intervals (intervals 1–10), *F* (9, 198) = 3.963, *p* < 0.001, $\eta_{p}^{2}$ = 0.153. A significant main effect was observed for exercise condition on %VO_2_peak in which BW-HIIE (77.4 ± 4.0%) was significantly lower than RUN-HIIE (88.0 ± 2.9%); *F* (1, 22) = 25.545, *p* < 0.001, $\eta_{p}^{2}$ = 0.537. A significant main effect was observed for time in which %VO_2_peak significantly increased across all intervals (1–10), *F* (9,198) = 10.785, *p* < 0.001, $\eta_{p}^{2}$ = 0.329.

### Heart Rate

Figure 2B displays the effect of high-intensity intervals 1–10 on %HRpeak. No significant interaction effect was observed on %HRpeak for type of HIIE and sequence of exercise intervals (intervals 1–10), *F* (9, 198) = 1.353, *p* = 0.212, $\eta_{p}^{2}$ = 0.058. A significant main effect for exercise condition was observed on %HRpeak in which BW-HIIE (88 ± 2%) was significantly lower than RUN-HIIE (93 ± 1%), *F* (1, 22) = 4.668, *p =* 0.042, $\eta_{p}^{2}$ *=* 0.175. A significant main effect for time was observed in which %HRpeak significantly increased across all intervals (1–10) *F* (9,198) = 13.254, *p* < 0.001, $\eta_{p}^{2}$ = 0.376. Figure 2C displays average HR attained for BW-HIIE and RUN-HIIE. No significant interaction effect was observed on average HR for type of HIIE and sequence of exercise intervals (intervals 1–10), *F* (9,198) = 1.40, *p* = 0.191, $\eta_{p}^{2}$ = 0.06. A significant main effect was observed for condition in which BW-HIIE (166 ± 3 bpm) was significantly lower than RUN-HIIE (175 ± 1 bpm), *F* (1,22) = 5.85, *p* = 0.24, $\eta_{p}^{2}$ = 0.210. A significant main effect was observed for time in which average heart HR significantly increased across all intervals (1–10), *F* (9,198) = 13.60, *p <* 0.001, $\eta_{p}^{2}$ = 0.382.

### Blood Markers and Perceptual Responses

#### B_lac_ and CK

**Figure 3:**
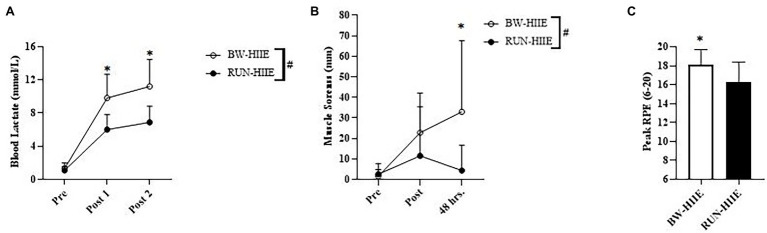
**(A)** Blood lactate (B_lac_) responses of BW-HIIE and RUN-HIIE before exercise (Pre), during 2 min of passive rest between set 1 and 2 (Post 1), and during 4 min of passive rest after set 2 (Post 2). **(B)** Perceived muscle soreness before exercise (Pre), upon completion of BW-HIIE and RUN-HIIE (Post), and 48 h post-exercise (48 h.). **(C)** Peak rating of perceived exertion (RPE) during BW-HIIE and RUN-HIIE. BW-HIIE = high-intensity interval exercise with bodyweight, RUN-HIIE = high-intensity interval exercise with treadmill, ^#^*p* < 0.001 BW-HIIE significantly higher than RUN-HIIE, and ^*^p < 0.05 BW-HIIE significantly higher than RUN-HIIE. Data are expressed as mean ± SD.

Figure 3A displays the effect of different types of HIIE (BW-HIIE and RUN-HIIE) and time (pre-HIIE, post-set 1, and post-set 2) on B_lac_. A significant interaction effect was observed for type of HIIE exercise and time, *F* = (2,44) = 11.433, *p* < 0.001, $\eta_{p}^{2}$ = 0.342. A significant main effect was observed for exercise condition on B_lac_ in which B_lac_ was significantly higher for BW-HIIE (11.2 ± 3.2 mmol/l) than RUN-HIIE (6.9 ± 2.0 mmol/l), *F* (1,22) = 17.634, *p* < 0.001, $\eta_{p}^{2}$ = 0.445. No significant differences in B_lac_ were observed at baseline for BW-HIIE and RUN-HIIE, *p* > 0.05, *g* = 0.379; B_lac_ was significantly higher after set 1 for BW-HIIE compared to RUN-HIIE, *p* < 0.001, *g* = 1.626 and after set 2 for BW-HIIE compared to RUN-HIIE, *p* < 0.001, *g* = 1.615. A significant main effect was observed for time in which B_lac_ significantly increased across all time points, *F* (2,44) = 166.813, *p* < 0.001, $\eta_{p}^{2}$ = 0.883. No significant interaction effect was observed on blood CK concentration for the type of HIIE and time (pre-HIIE, post-HIIE, and 48 h post-HIIE) *F* (1, 22) = <0.001, *p* = 1.0, $\eta_{p}^{2}$ = <0.001. No significant main effect was observed for exercise condition on CK for BW-HIIE (153.21 ± 90.88 U/l) and RUN-HIIE (154.97 ± 83.79 U/l), *F* (1, 22) = 0.003, *p* = 0.959, $\eta_{p}^{2}$ = <0.001. No significant main effect was observed for time on blood CK, *F* (1,22) = 1.445, *p* = 0.242, $\eta_{p}^{2}$ = 0.062.

#### Perceived Muscle Soreness

Figure 3B displays the effect of different types of HIIE (BW-HIIE and RUN-HIIE) and time (pre-HIIE, post-HIIE, and 48 h post-HIIE) on perceived muscle soreness. A significant interaction effect was observed for type of HIIE and time (pre-HIIE, post-HIIE, and 48 h post-HIIE) on perceived muscle soreness, *F* (2, 44) = 3.776, *p* = 0.031, $\eta_{p}^{2}$ = 0.147. A significant main effect for condition was observed in which muscle soreness was significantly higher for BW-HIIE (19.1 ± 13.0 mm) than RUN-HIIE (6.1 ± 3.8 mm); *F* (1, 22) = 6.434, *p* = 0.019, $\eta_{p}^{2}$ = 0.226. No differences were observed for muscle soreness at baseline for BW-HIIE and RUN-HIIE, *p* > 0.05, *g* = −0.224 or post-exercise for BW-HIIE and RUN-HIIE, *p* = 0.254, *g* = 0.498. Muscle soreness was significantly higher 48 h post-exercise for BW-HIIE exercise than RUN-HIIE, *p* < 0.001, *g* = 1.058. A significant main effect for time was observed in which muscle soreness increased across all time points, *F* (2,44) =5.722, *p* = 0.006, $\eta_{p}^{2}$ = 0.206.

#### RPE and PACES

Figure 3C displays average peak RPE for BW-HIIE compared to RUN-HIIE. Higher peak RPE values were reported during BW-HIIE compared to RUN-HIIE, *p* = 0.016, *g* = 1.034. Similarly, the average RPE reported during BW-HIIE (16 ± 2) was significantly higher than during RUN-HIIE (14 ± 2), *p* = 0.003, *g* = 0.996. Regarding the active recovery periods, there was no significant difference in average RPE for BW-HIIE (11 ± 1) compared to RUN-HIIE (11 ± 1), *p* ≥ 0.05, *g* = 0.0, or peak RPE for BW-HIIE (13 ± 1) compared to RUN-HIIE (13 ± 1), *p* ≥ 0.05, *g* = 0.0. Additionally, no significant differences in PACES scores were found between BW-HIIE (97.0 ± 17.6) and RUN-HIIE (91.9 ± 22.6; *p* = 0.255, *g* = 0.243).

## Discussion

In the present study, we compared the acute physiological and perceptual responses between bodyweight exercises performed at an ‘all-out’ effort (BW-HIIE) and constant load treadmill running HIIE (RUN-HIIE). The major findings were that RUN-HIIE elicited greater cardiorespiratory strain (%VO_2_peak and %HRpeak) compared to the BW-HIIE; however, BW-HIIE elicited greater B_lac_, RPE, and delayed muscle soreness, respectively. The protocols similarly elicited no differences in CK concentration or enjoyment as assessed *via* PACES. Both BW-HIIE and RUN-HIIE elicited significant but somewhat distinct cardiorespiratory, B_lac_, and perceptual responses associated with strenuous exercise. The acute cardiometabolic responses of BW-HIIE may confer physiologic benefits of traditional HIIE and cross-training (i.e., muscular strength and endurance training) without the need for specialized equipment.

Although VO_2_ analysis is limited when investigating high-intensity exercise due to the contribution of non-oxidative ATP resynthesis (Panissa et al., 2018), results from the current study indicated that an acute bout of RUN-HIIE at a fixed intensity elicits a greater VO_2_ response (~88% VO_2_peak) compared to ‘all-out’ BW-HIIE (~77% VO_2_peak). In contrast, B_lac_ was ~3.8 mmol/l and ~ 4.3 mmol/l greater for BW-HIIE during and after exercise, respectively, compared to RUN-HIIE. In this context, we speculate that RUN-HIIE activates proportionally more oxidative muscle fibers and BW-HIIE activates proportionally more non-oxidative muscle fibers. Calisthenic exercises such as squat jumps and lunges involve more prominent eccentric muscle contractions resulting in a lower oxygen demand when compared to traditional forms of endurance exercise (Abbott et al., 1952; Douglas et al., 2017); therefore, the lower VO_2_ response with BW-HIIE may be related to the potentially greater eccentric components involved with the exercises. Further, the intensity and complexity of the movements involved with the calisthenic exercises likely contributed to reduced cardiorespiratory strain (i.e., brief periods of pausing while transitioning to different movements and less frequent muscle contractions) and mismatch between loads (i.e., fixed load RUN-HIIE).

Our results align with previous studies comparing traditional forms of cycle ergometry HIIE to calisthenic-based HIIE (Gist et al., 2014; Riegler et al., 2017). Although, the cardiorespiratory responses of our BW-HIIE were closer to those of Gist et al. (2014) who found 30s of repeated burpees elicited approximately 78%VO_2_peak and 85%HRpeak, compared to Riegler’s 30s of mixed upper and lower body calisthenics which elicited slightly higher cardiorespiratory responses (81% VO_2max_ and 90% HRmax, respectively; Riegler et al., 2017). In contrast to these studies (Gist et al., 2014; Riegler et al., 2017), our combination of calisthenic exercises resulted in greater B_lac_ and RPE responses compared to traditional RUN-HIIE. The combination of calisthenics used by Riegler et al. (2017) (i.e., 30s bursts of plyometrics, upper/lower body exercises, and isometrics) may have allowed participants to keep a steady pace and consistent intensity, whereas our calisthenic exercises were predominantly lower body plyometrics and performed for 60 s. Therefore, the duration of our calisthenics and localized fatigue likely played a prominent role in observed differences between the acute responses in our study and previous ones (Gist et al., 2014; Riegler et al., 2017). Further, the cardiorespiratory fitness levels of our participants and those of Gist et al. (2014) were slightly more elevated than Riegler et al. (2017) which may have contributed to lower relative HR and VO_2_peak values. Nonetheless, our findings further support those of Gist et al. (2014) and Riegler et al. (2017) by demonstrating calisthenics performed at an ‘all-out’ effort can elicit vigorous cardiorespiratory and perceptual responses as defined by the broad classifications set by ACSM [i.e., 64–90% of maximal oxygen uptake, 77–95% of maximum heart rate, or a rating of perceived exertion of somewhat hard to very hard (RPE 14–17)] (Garber et al., 2011; American College of Sports Medicine, 2018).

With respect to the heart rate response between BW-HIIE and RUN-HIIE, we found %HRpeak and average HR were significantly lower during BW-HIIE compared to RUN-HIIE. However, less of a difference occurred in the HR response (%HRpeak ~5% lower for BW-HIIE versus RUN-HIIE) than the VO_2_ response (%VO_2_peak ~ 11% lower for BW-HIIE versus RUN-HIIE). The mismatch between VO_2_ and HR responses might be explained by the characteristics of the skeletal muscle contractions being performed and the type of load imposed on the heart. We speculate that BW-HIIE required less frequent and more forceful muscle contractions which would be expected to produce brief instances of elevated load on the heart (afterload), whereas RUN-HIIE required more frequent muscle contractions and a more constant load on the heart, allowing greater autonomic control (i.e., sympathetic outflow) and VO_2_ attainment. For instance, a previous study found BW-HIIE elicited greater sympathetic activity assessed *via* decreased heart rate variability and elevated catecholamines (epinephrine and norepinephrine) compared to treadmill running, despite both protocols being performed at similar exercise intensities (~90% HRmax; Kliszczewicz et al., 2016). Additionally, the greater recruitment of more non-oxidative muscle fibers may have resulted in subsequent accumulation of metabolites (i.e., hydrogen ions, inorganic phosphate, and lactate; Collins et al., 1991) which increases HR through the stimulation of Group III/IV afferents (Amann et al., 2015). Further, the higher RPE we observed with BW-HIIE may be indicative of sensations felt from greater localized stress on the musculoskeletal system (Ekblom and Golobarg, 1971; Pandolf and Noble, 1973; Brewer et al., 2021). These factors may have impacted the self-selected ‘all-out’ pace for participants during the BW-HIIE protocol. For reasons mentioned above, we speculate the greater localized muscle strain induced by BW-HIIE and subsequent adjustments in self-paced efforts may have impacted VO_2_ and HR kinetics.

In relation to exercise-induced muscle soreness, we observed a higher perceived muscle soreness at 48 h after BW-HIIE compared to RUN-HIIE. This difference in muscle soreness may be related to greater eccentric muscle contractions involved with BW-HIIE (Lieber and Fridén, 1999). Surprisingly, we did not observe differences in blood CK between pre- and 48 h post-exercise or between BW-HIIE and RUN-HIIE. Although the participants of the present study were physically active and have participated in HIIE and plyometric-based calisthenics, we were expecting an increase in blood CK concentration in response to the exercise protocols. Previous studies have noted a delay in appearance of CK in blood with comparisons between predominantly concentric versus eccentric type exercise with rises occurring 24 h post-concentric exercise and 4–7 days post-eccentric exercise (Newham et al., 1986). In addition, it could be postulated that the potential for greater localized fatigue of muscle groups used in BW-HIIE, and the wall-sit position used for the pain scale assessment may have presented large differences in perceived soreness without necessarily reflecting differences in blood CK. However, our results on perceived muscle soreness should be interpreted with caution given the large standard deviation between the protocols. Finally, the PACES did not reveal a difference in the level of enjoyment between BW-HIIE and RUN-HIIE. This result is similar to Olney et al. (2018) who found no differences in mean PACES between acute moderate-intensity continuous cycling (81.4 ± 7.7) and three different acute cycling-based HIIE protocols (i.e., low-volume HIIT: 85.6 ± 18.0, high-volume HIIT: 85.2 ± 17.3, and sprint interval training 87.6 ± 21.2; Olney et al., 2018).

There are several limitations which should be noted regarding the present study. The BW-HIIE protocol used a variety of exercises in a specific order which resulted in heterogeneous physiological and perceptual response. Although both BW-HIIE and RUN-HIIE involved predominantly lower body exercises, it is difficult to make these work-matched; this limits direct comparisons between the protocols. Further, the participants were instructed to perform the BW-HIIE at an ‘all-out’ self-paced intensity and the RUN-HIIE at a fixed intensity based on Vmax attained during a progressive maximal exercise test. This difference forced the participants in the RUN-HIIE to maintain a more even pace and may have contributed to a higher VO_2_ and different perceptual responses. Future investigations comparing the acute responses of calisthenics performed in an ‘all-out’ effort to ‘all-out’ running in which the individual controls stride, speed, and power output (e.g., self-propelled treadmill runners or overground fixed distance sprints) are warranted. Lastly, this study included a small sample of recreationally trained individuals (VO_2_peak norms ≥90th *n* = 7; 70th–75th *n* = 3; 55th–65th *n* = 2; American College of Sports Medicine, 2018) which limits the application of our findings and prompts further investigations as to how BW-HIIE can be modified for the general public and clinical populations.

## Conclusion

Our results suggest calisthenic-based ‘all-out’ exercises using a mix of aerobic and lower body muscle strengthening/plyometric type movements can elicit vigorous cardiorespiratory, perceptual, and blood lactate responses. From a practical standpoint, calisthenic-based high-intensity exercise may elicit cardiometabolic benefits associated with traditional high-intensity treadmill running without the financial burden and need of a treadmill.

## Data Availability Statement

The raw data supporting the conclusions of this article will be made available by the authors, without undue reservation.

## Ethics Statement

The studies involving human participants were reviewed and approved by The University of New Mexico Innovative Solutions for Compliance and Research Management (IRB). The participants provided their written informed consent to participate in this study.

## Author Contributions

All authors provided significant contributions to the article and none of the authors had a conflict of interest. GB, JD, and FA were involved with all aspects of the study (study proposal, study design, data collection, data analysis, and manuscript preparation). ZM, DM, and JS were involved with data collection and data analysis. MS and JL were involved with data analysis and manuscript preparation. MD, AG, and FC were involved with manuscript preparation.

## Funding

This study was funded by the New Mexico NM-INBRE Developmental Research Project Program (DRPP) grant number P20GM103451.

## Conflict of Interest

The authors declare that the research was conducted in the absence of any commercial or financial relationships that could be construed as a potential conflict of interest.

## Publisher’s Note

All claims expressed in this article are solely those of the authors and do not necessarily represent those of their affiliated organizations, or those of the publisher, the editors and the reviewers. Any product that may be evaluated in this article, or claim that may be made by its manufacturer, is not guaranteed or endorsed by the publisher.
