# Supplementary material for: The Acute Physiological and Perceptual Responses Between Bodyweight and Treadmill Running High-Intensity Interval Exercises
Source: Front Physiol. 2022 Mar 10;13:824154. doi: 10.3389/fphys.2022.824154 (PMC8960724; doi:10.3389/fphys.2022.824154)
Supplement: Supplementary file 1 [file Table_1.pdf]

Supplementary Material

Supplementary Tables

Table 1. Anthropometric and physiological data for female (n = 6) and male (n = 6) participants. Data are displayed as means ± standard deviation.

	Female (n=6)	Male (n=6)	All (N = 12)
Age (years)	30.5 ± 6.7	28.5 ± 3.7	29.5 ± 5.3
Height (cm)	161.8 ± 5.7	173.9 ± 7.5	167.9 ± 8.9
Weight (kg)	60.5 ± 5.1	81.3 ± 14.5	70.9 ± 15.0
BF (%)	20.1 ± 3.4	10.0 ± 3.8	15.0 ± 6.3
VO_2peak (mL· min^{-1}·kg^{-1})	43.2 ± 3.6	54.3 ± 3.4	48.7 ± 6.7

N= 12. cm= centimetres, kg = kilograms; BF (%) = body fat percentage; VO_2peak = peak oxygen consumption; ml = milliliters, min = minute

Table 2. Repetitions performed during BW-HIIE (N = 12).

Exercise	Set 1	Set 2	*P*-value	*g*
High knees	185 ± 16	183 ± 22	0.770	0.100
Squat jumps	50 ± 6	47 ± 8	0.022*	0.410
Scissor Jacks	154 ± 13	153 ± 10	0.481	0.083
Jumping Lunges	55 ± 10	51 ± 11	0.527	0.367
Modified Burpees	24 ± 3	22 ± 4	0.076	0.546

g = Hedges's *g*, **P* <0.05 significantly lower compared to the second set.
