# Supplementary figures and images for: The Acute Physiological and Perceptual Responses Between Bodyweight and Treadmill Running High-Intensity Interval Exercises
Source: Front Physiol. 2022 Mar 10;13:824154. doi: 10.3389/fphys.2022.824154 (PMC8960724; doi:10.3389/fphys.2022.824154)

## Supplementary Material

Figure 1.

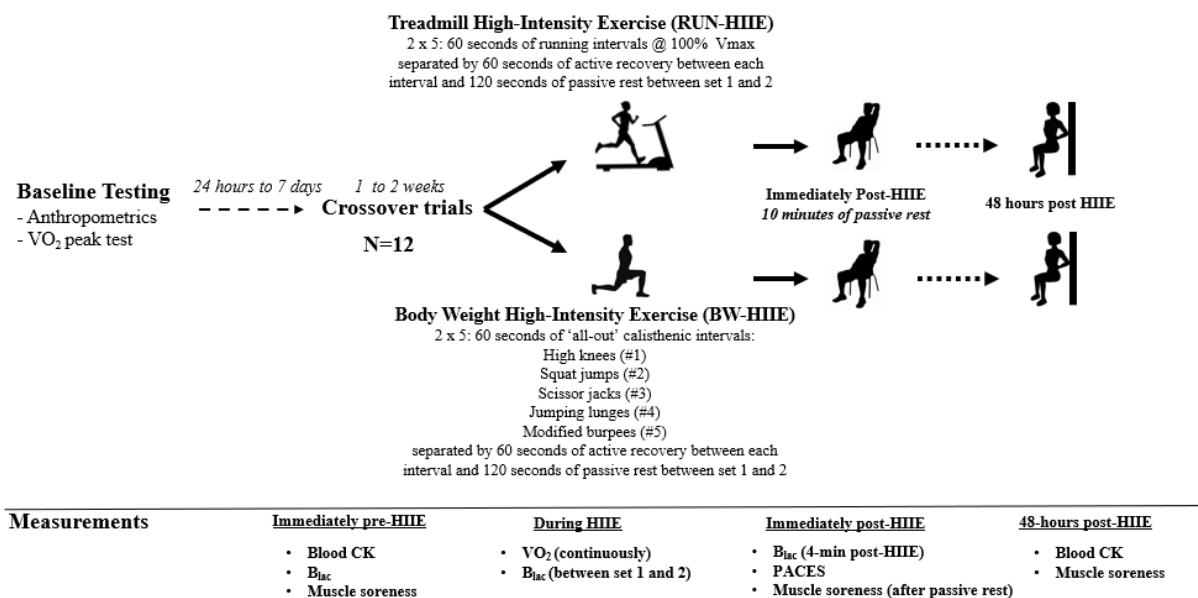

Figure 2.

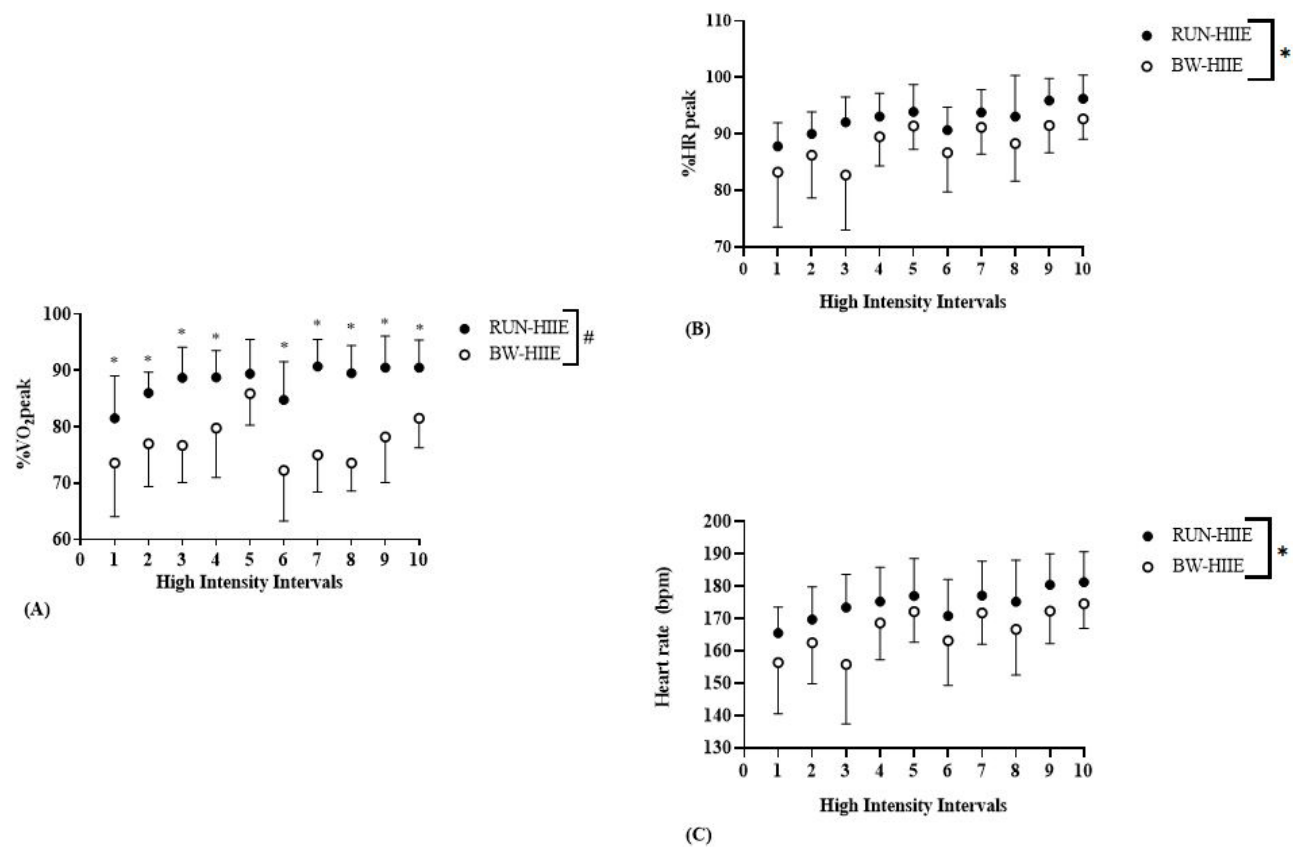

Figure 3.

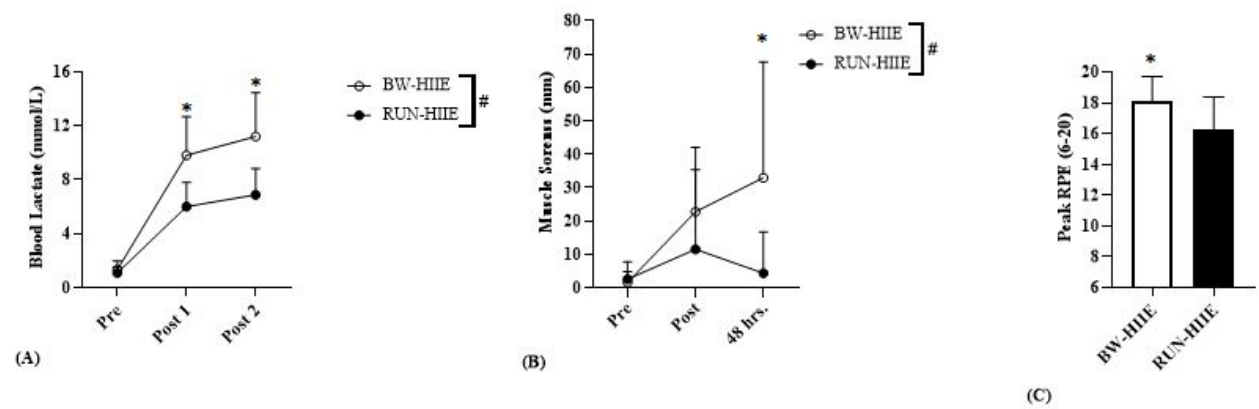

Supplement: Supplementary file 2 [file Image_1.pdf]
